# Evaluation of an ensemble of genetic models for prediction of a quantitative trait

**DOI:** 10.3389/fgene.2014.00474

**Published:** 2015-01-13

**Authors:** Jacqueline N. Milton, Martin H. Steinberg, Paola Sebastiani

**Affiliations:** ^1^Department of Biostatistics, School of Public Health, Boston UniversityBoston, MA, USA; ^2^Department of Medicine, School of Medicine, Boston UniversityBoston, MA, USA

**Keywords:** genetic risk prediction, genetic risk score, ensemble-based classifiers, bagging predictors, prediction accuracy

## Abstract

Many genetic markers have been shown to be associated with common quantitative traits in genome-wide association studies. Typically these associated genetic markers have small to modest effect sizes and individually they explain only a small amount of the variability of the phenotype. In order to build a genetic prediction model without fitting a multiple linear regression model with possibly hundreds of genetic markers as predictors, researchers often summarize the joint effect of risk alleles into a genetic score that is used as a covariate in the genetic prediction model. However, the prediction accuracy can be highly variable and selecting the optimal number of markers to be included in the genetic score is challenging. In this manuscript we present a strategy to build an ensemble of genetic prediction models from data and we show that the ensemble-based method makes the challenge of choosing the number of genetic markers more amenable. Using simulated data with varying heritability and number of genetic markers, we compare the predictive accuracy and inclusion of true positive and false positive markers of a single genetic prediction model and our proposed ensemble method. The results show that the ensemble of genetic models tends to include a larger number of genetic variants than a single genetic model and it is more likely to include all of the true genetic markers. This increased sensitivity is obtained at the price of a lower specificity that appears to minimally affect the predictive accuracy of the ensemble.

## Introduction

Genome-wide association studies (GWAS) have been used extensively to examine the association between common single nucleotide polymorphisms (SNPs) and disease phenotypes. While many of these studies have successfully found genetic variants that have highly significant associations with phenotypes, typically their effect sizes are small and the predictive power is limited. In order to build genetic prediction models with hundreds of SNPs, investigators often combine multiple SNPs into a genetic score that is used as a single covariate (Meigs et al., 2008; Purcell et al., 2009; Paynter et al., 2010; Sebastiani et al., 2012b; Kundu et al., 2014). The genetic score is built by adding the number of alleles from a list of SNPs that are found associated with consistent changes in the phenotype, often from the result of a GWAS or previously published work, but one of the difficulties in developing a genetic score for phenotype prediction is the determination of the optimal number of SNPS to be used. Including too few variants could limit the prediction accuracy, while including too many genetic variants could introduce too many false positives SNPS and therefore impact the accuracy of the prediction model.

Ensemble methods have been utilized to address these challenges (Breiman, 1996), and we introduced an ensemble of Bayesian classification rules for prediction of qualitative phenotypes using genetic data in Hartley et al. (2012), Sebastiani et al. (2012a), Hartley and Sebastiani (2013). In Milton et al. (2014), we generalized the approach to prediction of a quantitative phenotype: fetal hemoglobin level in sickle cell anemia patients. In this paper we formally describe the approach and use simulations to compare the results of the ensemble-based method vs. using a single “best” model, in the specific case of unweighted genetic score. We show that an ensemble of genetic models is more robust to the inclusion of genetic variants that are falsely associated with the phenotype than using a single model.

## Materials and methods

### Development of genetic scores and genetic prediction models

The method assumes that there is a list of *S* SNPs ordered by decreasing statistical significance that result from a GWAS. We assume that these SNPs are not in linkage disequilibrium. Let the “risk allele” of each SNP be the allele that is associated with an increase in the quantitative trait using an additive genetic model. Therefore, an individual *i* can carry 0, 1, or 2 risk alleles for each SNP *j* that we denote by *R_i,j_*. For a fixed number of SNPs *N*, the genetic score for individual *i* is computed by adding the number of risk alleles of the *N* SNPs as follows:

$$GS_{i,N}=\;\sum_{j=1}^{N}R_{i,j}$$

This genetic score *GS_i,N_* based on *N* SNPs is used as a covariate in the linear regression model:

(1) $$E(y_{i,N})=\;\beta_{0,N}+\beta_{1,N}\;GS_{i,N}$$

where *y_i,N_* is the phenotype of the *i*th individual and the regression coefficients β_0,*N*_, β_1,*N*_ can be estimated using the Maximum Likelihood (ML) method. The prediction of the phenotype for an individual with genetic score *GS_i,N_* is then provided by the formula:

(2) $${\hat{y}}_{i,N}=\;\hat{\beta_{0,N}}+\hat{\beta_{1,N}}\;GS_{i,N}$$

where $\hat{\beta_{0,N}}$ and $\hat{\beta_{1,N}}$ are the ML estimates of the regression coefficients. As the number *N* of SNPs included in the genetic score varies, one can compute different genetic scores for each individual and therefore different genetic prediction models. The challenge is to choose the best number of SNPs to be included in the genetic score for optimal prediction. A simple approach is to randomly divide the data into a training set and a test set, use the training set to generate cumulative genetic scores by adding one SNP at a time from the sorted list of SNPs so that *GS*_*i,N*+1_ = *GS*_*i,N*_ + *R*_*i,N*+1_ for *N* = 1,…,*S*, and use these *S* models to predict the outcome in the test set. The model with the largest correlation between predicted and observed phenotype in the test set will identify the best genetic score, and therefore the best number of SNPs. In this sampling strategy, “single split,” the dataset is split into training and test sets only once.

### Cross validation to choose the optimal *N*

K-fold cross validation (CV) can also be used to determine the optimal number of SNPs (Alsultan et al., 2011). In K-fold cross validation, the dataset is randomly partitioned into K equally sized, non-overlapping datasets. Iteratively, one partition is reserved as a test set and K-1 of the K partitions are merged into a training dataset that is used to develop *S* genetic models, one for each of the genetic scores *GS_i,N_*, *N* = 1,…,*S*. The *S* genetic models are used to predict the outcome in the test set and the model with the largest correlation between predicted and observed outcome is selected as the most predictive model.

### Development of ensemble of genetic prediction models

Phenotype prediction can also be accomplished by using an ensemble of genetic models (Hartley et al., 2012; Sebastiani et al., 2012a). The idea of the ensemble methodology is to build a predictive model by combining predictions from multiple models. Here, we propose an ensemble of *M* cumulative genetic models in which the predicted value of a phenotype is computed as the average prediction from *M* genetic models as follows:

(3) $${\bar{\hat{y}}}_{i,M}=\;\frac{1}{M}\;\sum_{N=1}^{M}{\hat{y}}_{i,N.}$$

In Equation (3) ${\hat{y}}_{i,N}$ is the prediction from the model with genetic score *GS_i,N_* for individual *i* and the genetic scores are cumulatively built by adding one SNP at a time from the sorted list of SNPs so that *GS*_*i,N*+1_ = *GS*_*i,N*_ + *R*_*i,N*+1_. Therefore, *M* represents the number of models in the ensemble as well as the overall number of SNPs used for prediction. To choose the number *M*, the data can be randomly divided into a training set and a test set, and the prediction accuracy of the ensemble of increasing number of genetic prediction models (*M* = 1,…,*S*) generated in the training set can be evaluated in the independent test set to identify the ensemble of *M* models with best prediction (the model with the largest correlation between the predicted and observed phenotype) (Mevik et al., 2004).

### Simulations

We tested the prediction accuracy of the single genetic model and the ensemble of genetic models on simulated data. The following simulation scheme was adapted from Yip and Lange (2011), Bae et al. (in press).

1. *S* = 1000 biallelic SNPs were generated with minor allele frequency (MAF) that followed a uniform distribution in the interval (0.05, 0.50). The 0.05 cutoff of the uniform distribution was used to mimic a quality control process where SNPs with a MAF < 0.05 are removed from a GWAS dataset. The genotypes *G_i,k_* where *G_i,k_* is the additive genotype coding for the *i*^th^ individual at the *k^th^* causal SNP, were generated using a multinomial distribution, assuming Hardy–Weinberg equilibrium for each SNP so that, for each allele frequency *p*, genotypes were simulated in proportions *p*^2^, 2*p*(1 - *p*) and (1 - *p)^2^*. A sample of 1000 individuals was generated for each simulated data set.
2. The phenotype was generated from a linear regression model with *m* = 5, 10, and 30 causal SNPs (out of *S* = 1000) with a total variability σ^2^_*Total*_ = 1. Here we define a causal SNP to be a SNP truly associated with the phenotype (a true positive). We chose three different levels of heritability: low (*h*^2^ = 0.20), medium (*h*^2^ = 0.40), and high (*h*^2^ = 0.60), and for each *h*^2^ we defined the effect size *a_k_* for each causal SNP, under a strictly additive model, as:
  (4) $$a^{2}{}_{k}=\;\frac{h_{k}^{2}\sigma {\;}_{Total}^{2}}{2p_{k}(1-p_{k})}$$
3. The formula in Equation (4) was described in Yip and Lange (2011), Bae et al. (in press), and σ^2^_*Total*_ is the total phenotypic variability, *p_k_* is the MAF of the *k^th^* causal SNP, $h_{k}^{2}=\frac{{\text{h}}^{2}}{m}$ is the heritability of the *k^th^* causal SNP, and *m* is the number of causal SNPs. The effect size in Equation (4) assumes that all causal SNPs contribute to the total heritability by an equal amount. For each causal SNP, we randomly drew $y_{i,k}\;\sim \;N\left(a_{k}G_{i,k},\frac{\sigma_{Total}^{2}}{m}\right)$. The phenotype was then computed as follows:
  $$y_{i}=\;\sum_{k=1}^{m}y_{i,k}$$
  resulting in $y_{i}\;\sim \;N\left(\sum_{k=1}^{m}a_{k}G_{i,k},\sigma_{Total}^{2}\right)$.
4. The 1000 individuals in each simulated data set were randomly separated into a set of 900 individuals (discovery dataset) and 100 individuals (test dataset) that were used for model building and testing.

This simulation procedure was used to generate 1000 data sets for each combination of heritability and number of causal SNPs. A single SNP analysis was performed in each discovery dataset for all of the SNPs that were then sorted by order of statistical significance. Cumulative genetic scores were then computed for each of the 900 individuals in the training set by adding one SNP at a time from the sorted list of 1000 SNPs (sorted in order of decreasing significance), thus producing 1000 genetic scores for each subject, in each group, for each simulated dataset. We then generated genetic prediction models using the genetic scores as the covariate as shown in Equation (1) and the 1000 genetic models estimated from the training set in each simulation were used to predict the phenotype for the test dataset of 100 individuals using the formula shown in Equation (2). Ensembles of these genetic prediction models were also used to predict the phenotype for the test dataset as shown in Equation (3). The Pearson correlation between the predicted and observed phenotype was computed for all these models and the model with the highest correlation between observed and predicted values in the test set was selected as the most predictive.

We also randomly divided each of the 1000 simulated set into 10 partitions and used 10 fold CV to select the most predictive genetic prediction model in each simulated data set. For each of the 10 folds the correlation between the observed and predictive phenotype was computed for all genetic models. The results from the 10 folds were then averaged to produce a single estimation of the correlation between the observed and predicted phenotype for each genetic model. The model with the highest correlation was chosen to be the model with the optimal number of SNPs.

We summarize the results by the correlation between observed and predicted values using the single and ensemble of genetic models, the number of overall SNPs and the proportion of causal SNPs included in each selected model.

## Results

**Figure 1:**
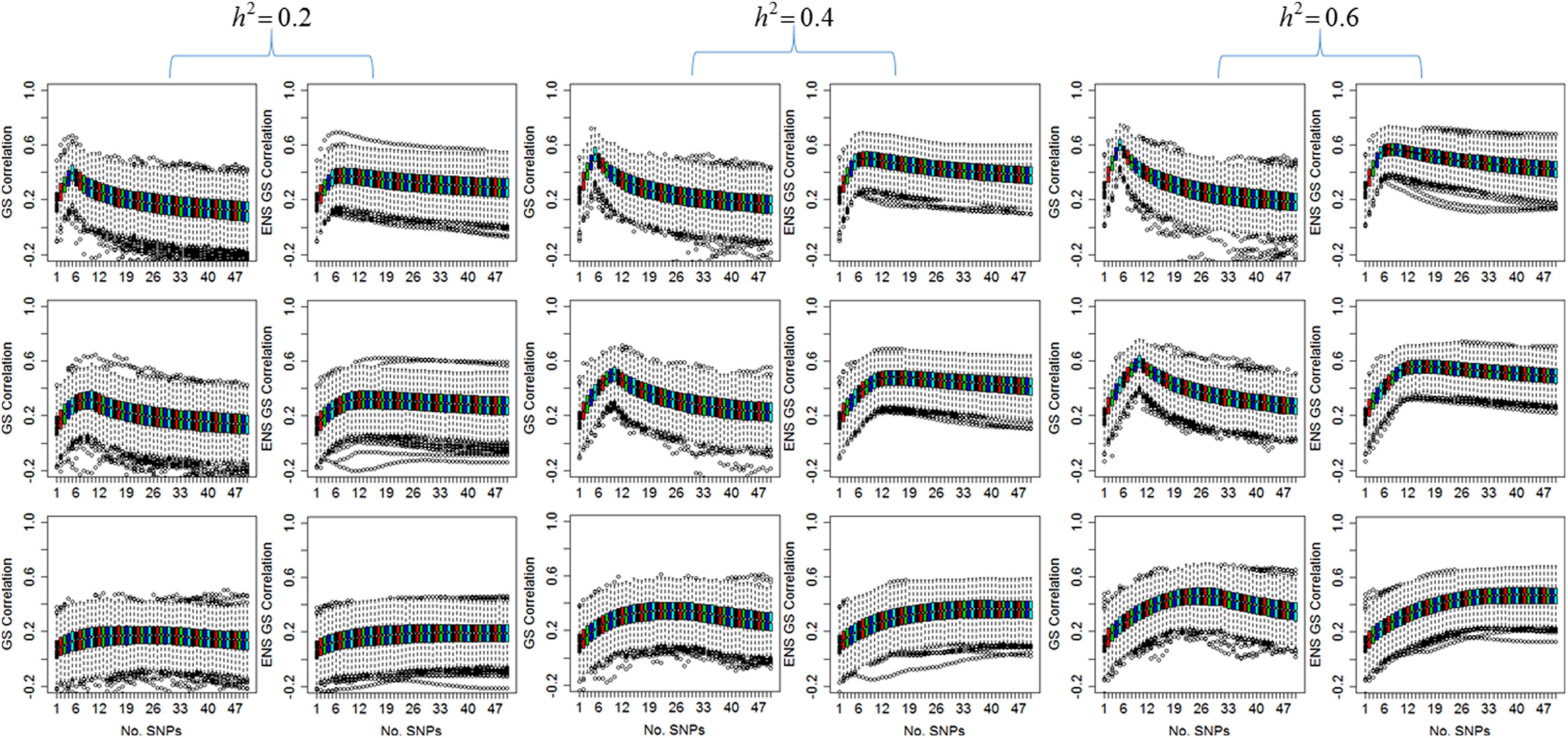
**Distribution of the correlation between observed values and values predicted by the single genetic model (GS) and the ensemble of genetic models (ENS GS)**. The side by side boxplots display the correlation between the observed and predicted phenotype in the 1000 simulated test data vs. the number of SNPs in the single genetic model (GS: odd columns) and the ensemble of genetic models (ENS GS: even columns) for increasing heritability (*h*^2^). **Row 1**: five causal SNPs; **row 2**: 10 causal SNPs; **row 3**: 30 causal SNPs.

Figure 1 displays the distribution of the correlation between the predicted and the observed values for the single genetic prediction model (GS correlation) and the ensemble methods (ENS GS correlation) for increasing number of SNPs in the genetic score. The phenotype was simulated assuming five causal SNPs (row 1), 10 causal SNPs (row 2), and 30 causal SNPs (row 3), and increasing heritability, but with fixed phenotypic variability.

The most obvious result in Figure 1 is that, for fixed number of causal SNPs, the correlation between the observed and predicted phenotype increases as the heritability of the phenotype increases. This result is expected since the effects of the causal SNPs increase with increasing heritability and fixed variability, as shown in Equation (4), and therefore the causal SNPs are more likely to be found statistically significant and ranked high in the list of SNPs to be included in the genetic score.

Both the single genetic model and the ensemble of genetic models show that the prediction accuracy tends to initially increase as more and more SNPs are added to the model. The single genetic model has a faster rate of increase than the ensemble of models, and it reaches a peak of prediction accuracy followed by a decline when the genetic score includes too many SNPs. On the other hand, the ensemble of genetic models appears to require a few more SNPs than the single genetic model to reach a peak of prediction accuracy but the rate of decline is markedly slower relative to the single genetic model as more and more SNPs are added to the models. These results suggests that the best single genetic model, selected on the best predictive accuracy in the test set, should include a smaller number of SNPs than the best ensemble of genetic models.

Consistent with this observation, Table 1 reports summary statistics (median and interquartile range) of the number of SNPs selected using the single split for the single genetic model and for the ensemble of genetic models and shows that the best single genetic prediction model tends to include a smaller number of SNPs than the best ensemble of genetic models. This smaller number is close to the number *m* of causal SNPs used in the simulations when *m* = 5 or 10, but it is an underestimate when *m* = 30 and the severity of the under-estimation increases with smaller heritability. The best ensemble of genetic models, on the other hand, tends to include a number of SNPs that exceeds the number of causal SNPs.

**Table 1:** **Distribution of the number of SNPs included in the best single genetic model selected with the single split of the data (GS), the best ensemble of genetic models (ENS GS), and the best single genetic model selected using cross-validation (CV)**.

**SNP**	**Method**	***h*^2^**		
		**0.2**	**0.4**	**0.6**
5	GS	5 (5, 6)	5 (5, 5)	5 (5, 5)
	ENS GS	8 (6, 11)	8 (7, 10)	8 (7, 9)
	CV	5 (5, 6)	5 (5, 5)	5 (5, 5)
10	GS	9 (7, 11)	10 (9, 10)	10 (10, 10)
	ENS GS	14 (10, 21)	15 (13, 19)	16 (14, 19)
	CV	9 (7, 10)	10 (9, 10)	10 (10, 10)
30	GS	17 (8, 29)	23 (16, 29)	24 (20, 29)
	ENS GS	30 (11, 48)	37 (30, 49)	40 (34, 49)
	CV	15 (9, 24)	21 (16, 26)	23 (20, 27)

*Numbers in the table are median and interquartile range*.

**Figure 2:**
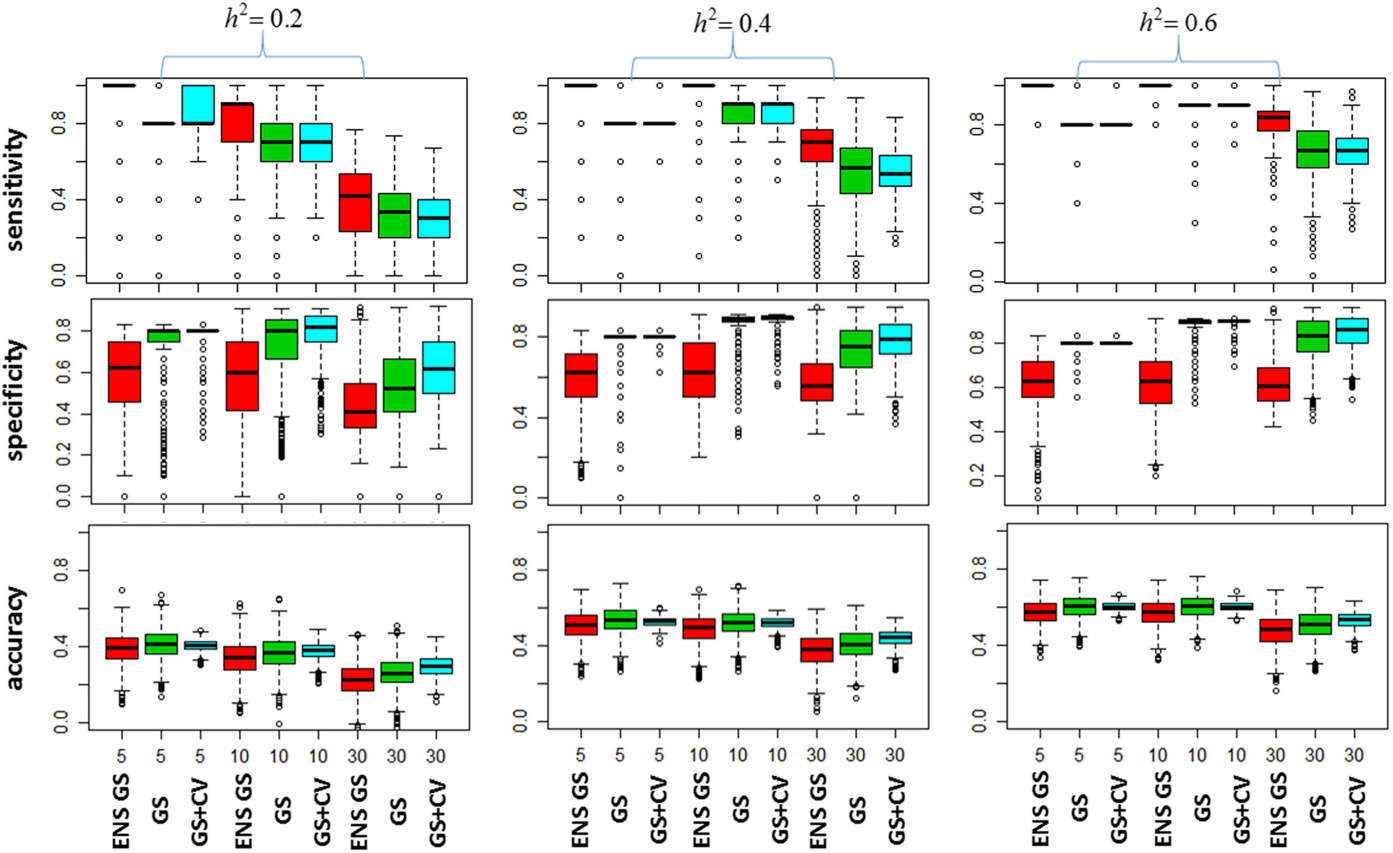
**Plots of the Model Selection Accuracy vs. Number of Causal SNPs**. The top panel displays side-by-side boxplots of the proportion of causal SNPs that were included in the most predictive models, for increasing number of SNPs (x-axis), and increasing heritability (*h*^2^). The mid panel displays side-by-side boxplots of the specificity of the most predictive models and the bottom panel displays summaries of the prediction accuracy of the same methods. ENS GS: the ensemble of genetic models selected using a single split of the data (red); GS: single genetic model selected using a single split of the data (green); GS + CV: single genetic model selected using 10 fold CV model (blue).

We next investigated how the different numbers of SNPs included in the best single genetic model and the best ensemble of genetic models affect the sensitivity of the methods, that is, the selection of true positive SNPs, and the prediction accuracy. Figure 2 (top panel) shows the sensitivity of the best ensemble of genetic models and of the best single genetic model. The sensitivity of the ensemble of genetic models is almost 100% with a small number of causal SNPs and decreases when the SNP effects become small, but it is higher than the sensitivity of the best single genetic model. The higher sensitivity comes at a price of lower specificity (Figure 2, mid panel) but the bottom panel of Figure 2 shows that the accuracy of the best ensemble of genetic models is only slightly inferior to the accuracy of the best single genetic model. Table 2 reports summary statistics (median and interquartile range) of the correlation between observed and predicted phenotypes and shows that the worst median loss of accuracy was about 15% when the heritability was low (*h*^2^ = 0.20), and the number of causal SNPs with large (*m* = 30), and it was within 5% in the other scenarios. The analysis suggests that the ensemble of genetic models is more likely to capture all causal SNPs than the single genetic model at the price of including some false positive SNPs, without substantially reducing the predictive accuracy.

**Table 2:** **Summary of the predictive accuracy of the best single genetic model selected with the single split of the data (GS), the best ensemble of genetic models (ENS GS), and the best single genetic model selected using cross-validation (CV)**.

**SNP**	**Method**	***h*^2^**		
		**0.2**	**0.4**	**0.6**
5	GS	0.41 (0.33, 0.39)	0.54 (0.49, 0.59)	0.60 (0.56, 0.65)
	ENS GS	0.39 (0.33, 0.44)	0.51 (0.46, 0.56)	0.58 (0.53, 0.62)
	CV	0.40 (0.38, 0.42)	0.53 (0.51, 0.54)	0.60 (0.59, 0.62)
10	GS	0.37 (0.31, 0.42)	0.52 (0.48, 0.57)	0.60 (0.56, 0.65)
	ENS GS	0.34 (0.28, 0.40)	0.49 (0.44, 0.54)	0.57 (0.52, 0.62)
	CV	0.38 (0.35, 0.41)	0.52 (0.50, 0.54)	0.60 (0.58, 0.62)
30	GS	0.26 (0.21, 0.31)	0.41 (0.35, 0.46)	0.51 (0.46, 0.56)
	ENS GS	0.22 (0.16, 0.28)	0.38 (0.32, 0.44)	0.48 (0.42, 0.53)
	CV	0.26 (0.21, 0.31)	0.44 (0.41, 0.47)	0.53 (0.51, 0.56)

*Numbers in the table are median and interquartile range*.

We also investigated whether a different selection of the best genetic model could produce a better inclusion of true positive SNPs. We used 10 fold CV, as described in the methods, to select the best single genetic model. The results in Table 1 show that CV produced best single genetic models that included a number of SNPs comparable to the strategy based on a single split of the data but the analysis of the accuracy and sensitivity of the models selected with 10 fold CV in Figure 2 and Table 2 suggests that the approach may be slightly less sensitive.

## Discussion

One of the major goals of GWAS was to identify genetic variants that are associated with disease or measures of disease severity in order to be used for personalized medicine. However, genetic models have been of limited utility and the selection of the best SNPs to be used for prediction is challenging (Schrodi et al., 2014). SNPs that reach genome-wide significance often only explain a small proportion of the variability of the phenotype and have little value for prediction. Many studies have shown the importance of including genetic variants beyond those that meet the genome-wide association threshold of 5 × 10^−08^ (Makowsky et al., 2011). However, many of these SNPs may be false positives and their inclusion in the prediction model can lower the accuracy of the prediction in new data (Kooperberg et al., 2009; Yang et al., 2010).

Our evaluation in simulated data suggests that using an ensemble of genetic models provides a more robust solution compared to selecting a single genetic model. The analysis showed that when there are only a few causal SNPs, both the single genetic model and the ensemble of genetic models perform similarly. However, when the number of causal SNPs increases or, equivalently, when the SNP effects are small, a single genetic model tends to underestimate the number of causal SNPs, while the ensemble of genetic models tend to include a larger proportion of the causal SNPs. This increased sensitivity of the ensemble is associated with only a slight decrease in the prediction accuracy. This analysis suggests that an ensemble of genetic models would be particularly useful to identify true positive SNPs that may be ignored in other analyses. The slow decline of prediction accuracy also makes the ensemble of genetic prediction models more insensitive to the inclusion of false positive SNPs.

In this manuscript we limited attention to the theoretical aspects of the ensemble of genetic models. We applied this methodology to real data in Milton et al. (2014) to predict fetal hemoglobin (HbF) levels in patients with sickle cell anemia using genetic data. To this end, we developed an ensemble of 14 genetic models in a discovery cohort of 841 sickle cell patients. The ensemble of 14 genetic models was used to predict the HbF levels of sickle cell anemia patients in 3 independent cohorts and reached a correlation ranging between 28% and 44% in the three studies. Consistently with the analyses described here, using the ensemble of genetic models produced more robust predictions than using a single genetic model.

Many statistical methods have been developed to model complex traits and increase the prediction accuracy including multivariate regression models and machine learning type approaches such as support vector machines (Wei et al., 2009; Wu et al., 2011), multifactorial dimensionality reduction (Moore et al., 2006), and Bayesian networks (Rodin and Boerwinkle, 2005; Sebastiani et al., 2005, 2012b; Jiang et al., 2011; Kang et al., 2011). Our analysis only compared the results of an ensemble of genetic prediction models to a single best genetic model. It will be interesting to extend the comparison to include these alternative approaches to generate genetic prediction models.

In our analysis we assumed that SNPs that enter the analysis are not in linkage disequilibrium, as this is a commonly made assumption (Paynter et al., 2010; Sebastiani et al., 2012b). The effect of including SNPs in linkage disequilibrium remains to be investigated. This work only examined genetic prediction models with a genetic score that weighs all risk alleles equally. Further work is needed to extend and evaluate this approach with more sophisticated genetic scores that use varying weights for the risk alleles (Kooperberg et al., 2009). In the genetic prediction of fetal hemoglobin that we reported in Milton et al. (2014) we investigated ensembles of genetic models with either unweighted or weighted genetic scores, and the results did not differ, although the SNPs included in the genetic models had standardized effects ranging between 3.7 and 12.5. However, it will be important to investigate how different choice of weights could improve the predictive accuracy of the ensemble.

Finally, the approach described made assumptions about the genetic modeling that limit the generalizability of this study. We assumed in our simulations that the genetic variants have an independent additive effect on the phenotype, and that they all explain the same proportion of variability. It will be interesting to examine the effect of other modes of inheritance, of non-uniform genetic effects, and of rare and common variants in future work.

### Conflict of interest statement

The authors declare that the research was conducted in the absence of any commercial or financial relationships that could be construed as a potential conflict of interest.
